# Analysis of the expression and prognosis for leukocyte immunoglobulin-like receptor subfamily B in human liver cancer

**DOI:** 10.1186/s12957-022-02562-w

**Published:** 2022-03-24

**Authors:** Jing Fan, Lili Wang, Miao Chen, Jiakang Zhang, Jiayan Li, Fangnan Song, Aidong Gu, Dandan Yin, Yongxiang Yi

**Affiliations:** 1 Clinical Research Center, The Second Hospital of Nanjing, Nanjing University of Chinese Medicine, Zhong Fu Road, Gulou District, Nanjing, Jiangsu People’s Republic of China 210003; 2 Nanjing University of Chinese Medicine, Han Zhong Road, Jianye District, Nanjing, Jiangsu People’s Republic of China 210029; 3 Department of Hepatobiliary Surgery, The Second Hospital of Nanjing, Nanjing University of Chinese Medicine, Zhong Fu Road, Gulou District, Nanjing, Jiangsu People’s Republic of China 210003

**Keywords:** Liver cancer, LILRB, Prognosis, Bioinformatics analysis, NK cells

## Abstract

**Background:**

Leukocyte immunoglobulin-like receptor subfamily B (LILRB), including 5 subtypes, is a group of inhibitory receptors in the immune system. The LILRB family is known to be involved in the tumor progression of various cancer types, especially liver cancer. However, the expression patterns and prognostic values of LILRB family members in liver cancer tissues remain unclear.

**Methods:**

We used the Oncomine database, GEPIA database, Kaplan–Meier Plotter, Timer, and TISIDB to assess the expression and prognostic value of the LILRB family in liver cancer patients. We also verified the expression of the LILRB family in tumor tissues and tumor-free liver tissues at the protein level by using immunohistochemistry. The STRING website was used to explore the interaction between the LILRB family and their related genes. The DAVID database was used to perform the gene ontology (GO) and Kyoto Encyclopedia of Genes and Genomes (KEGG) analyses. Flow cytometry was used to assess the infiltrated NK cells in liver cancer tissues.

**Results:**

Our study revealed that the mRNA expression of LILRB1, LILRB2, LILRB3, and LILRB5 was downregulated, while compared with normal tissues, the mRNA expression of LILRB4 was upregulated in liver cancer tissues. Survival analysis revealed that LILRB2 and LILRB5 mRNA expression levels were significantly positively associated with overall survival (OS) and disease-free survival (DSS) and that the mRNA expression of all LILRB family members was significantly positively correlated with recurrence-free survival (RFS) and progression-free survival (PFS). Next, we further found that the mRNA expression of all LILRB family members was significantly associated with the infiltration of B cells, CD8^+^ T cells, CD4^+^ T cells, macrophages, neutrophils, and dendritic cells in liver cancer. Finally, GO and KEGG analyses found that the LILRB family and its related genes were involved in antigen processing and presentation and natural killer cell-mediated cytotoxicity pathways.

**Conclusions:**

Our study suggested that LILRB family expression was associated with the prognosis of liver cancer patients and infiltrated immune cells. The LILRB family might be involved in antigen processing and presentation and natural killer cell-mediated cytotoxicity pathways.

**Supplementary Information:**

The online version contains supplementary material available at 10.1186/s12957-022-02562-w.

## Background

Primary liver cancer is the sixth most diagnosed cancer and the fourth deadliest cancer in the world [1]. Asia and Africa have the highest incidence rates of liver cancer [2, 3]. In China, liver cancer is the fourth most commonly diagnosed cancer [4]. In addition, there are more male patients than female patients. According to pathological types, primary liver cancer is divided into hepatocellular carcinoma (HCC), intrahepatic cholangiocarcinoma (ICC), and HCC-ICC [5]. Populations with hepatitis B virus (HBV) infection, hepatitis C virus (HCV) infection, nonalcoholic fatty hepatitis, and alcohol abuse have a high risk of liver cancers. Classic clinical screening methods are alpha-fetoprotein (AFP) and liver ultrasonography. Nuclear medical imaging and liver biopsy are used to estimate the prognosis of liver cancer patients. Considering the heterogeneity of liver cancer, the current method to screen or to predict the prognosis of liver cancer has limitations. Therefore, the identification of alternative and novel biomarkers is urgently needed for successful liver cancer treatment.

The leukocyte immunoglobulin-like receptor B (LILRB) family is a group of immune inhibitory receptors. The LILRB family consists of five members: LILRB1, LILRB2, LILRB3, LILRB4, and LILRB5. In an earlier study, the LILRB family was found to be expressed on many immune cells, such as dendritic cells, macrophages, B cells, T cells, and NK cells [6]. Our previous study also discovered that LILRB2 in CD1c^+^ myeloid DC subsets was remarkably increased in PBMCs of HCC patients and the microenvironment of liver cancer [7]. Engagement of LILRB1 and LILRB2 with its ligand HLA-G inhibits immune activation, resulting in indirect promotion of tumor development [8]. LILRB1 modulates the differentiation and function of dendritic cells, resulting in poor stimulating activity for primary and memory T cell proliferation [9]. LILRB1 also exhibited immune inhibition on NK cells [10]. Although the ligand for LILRB3 is not so clear, some studies suggested that LILRB3 was associated with cytokeratin-associated proteins exposed to necrotic cancer cells and might be involved in altering the immune responses within the tumor microenvironment [11]. LILRB5 is considered to be an orphan receptor but may be associated with mycobacteria to subvert immune responses [12, 13]. In addition to expression on immune cells, the LILRB family was also found to be expressed in multiple malignant cells, such as liver cancer and lung cancer [14, 15]. Cheng et al. [16] found that LILRB1 in hepatocarcinoma cells might integrate with SH2 domain-containing phosphatase-1 (SHP1) to exert an antitumor effect in liver cancer patients. In contrast, some studies have also shown that LILRB2 regulates tumor cell proliferation, invasion, and migration and promotes tumor progression in lung cancer [17, 18]. LILRB4 was specifically expressed in leukemia cells in monocytic acute myeloid leukemia but not in myelomonocytic acute myeloid leukemia [19].

Therefore, the researches about LILRB family members in cancer tissues are limited and the function of these is not clear. Moreover, the expression levels of the LILRB family and their correlation with clinical features and prognosis have not been reported completely, especially using bioinformatics analysis.

In this study, we aimed to explore the expression of LILRB family members in HCC tissues and find out the relationship between the LILRB family and clinical features and prognosis in HCC patients. Firstly, we used the Oncomine and gene expression profiling interactive analysis (GEPIA) databases to analyze the expression levels of the LILRB family in liver tumor tissues and their relationship with liver tumor stages. Moreover, immunohistochemistry was used to verify the results. Next, Kaplan–Meier Plotter was used to assess whether the LILRB family can be used to predict the prognosis of liver cancer patients. Then, we analyzed the correlation between LILRB family expression and immune cell infiltration by using tumor immune estimation resource (TIMER) and tumor and immune system interaction database (TISIDB). Finally, the retrieval of interacting genes with the LILRB family was exploded by using the search tool for the retrieval of interacting genes (STRING), and the potential signaling pathways related to the LILRB family were predicted through the Kyoto Encyclopedia of Genes and Genomes (KEGG) analysis.

## Materials and methods

### Oncomine database

The Oncomine platform (www.oncomine.org) is an online cancer microarray database. We used the Oncomine database to analyze the transcriptional levels of the LILRB family in different cancers, especially liver cancer. The conditions of the search filter were as follows: LILRB family gene name, cancer vs. normal analysis, threshold *p* value, 0.05, threshold fold change, 1.5, threshold gene rank, top 10%, and data-type mRNA.

### GEPIA database

GEPIA (gepia.cancer-pku.cn) 2.0 was developed by the Zhang laboratory from Peking University [20]. This online database served to analyze the RNA sequencing expression. All the data were from the TCGA and GTEx projects. In this study, GEPIA 2.0 was used to analyze tumor/normal differential expression and profiling about LILRB family members according to pathological stages.

### Kaplan–Meier Plotter

The associations between LILRB family members and survival were plotted as Kaplan–Meier curves using the Kaplan–Meier plotter (kmplot.com/analysis/) [21]. We used this database to analyze the correlation between LILRB family mRNA expression and the overall survival (OS), relapse-free survival (RFS), progression-free survival (PFS), and disease-free survival (DSS) of liver cancer patients. We also used this database to analyze the relationship between LILRB family mRNA expression and different tumor stages and the prognosis of liver cancer patients. We chose to select the best cutoff to split the LILRB family into a low-expression cohort and a high-expression cohort.

### TIMER

TIMER (cistrome.shinyapps.io/timer/) served as a comprehensive resource for the systematic analysis of immune infiltrates across diverse cancer types, including B cells, CD8^+^ T cells, CD4^+^ T cells, neutrophils, macrophages, and dendritic cells [22]. TIMER was used to analyze the relationship between LILRB family expression and tumor-infiltrating lymphocytes (TILs) in this study.

### TISIDB

TISIDB (cis.hku.hk/TISIDB/) developed by the Zhang laboratory in 2019 was a web portal for tumor and immune cell interactions [23]. All the data were from the PubMed database and The Cancer Genome Atlas (TCGA). We used this web portal to assess the relationship between LILRB family expression and various kinds of TIL subsets in this study.

### STRING database

STRING (string-db.org/cgi/input.pl) is an online database of known and predicted protein-protein interactions (PPIs) [24]. STRING was used to construct a PPIs network of the LILRB family. The condition of the minimum required interaction score was set as the highest confidence (0.900). Then, Cytoscape software V3.8.0 was employed to analyze the relationship between LILRB family members and their related genes.

### DAVID database

The Database for Annotation, Visualization and Integrated Discovery (DAVID) database (david.ncifcrf.gov/home.jsp) was used to carry out gene ontology (GO) and Kyoto Encyclopedia of Genes and Genomes (KEGG) analysis [25]. The R language was used to process the results. A false discovery rate (FDR) < 0.05 was considered to be significant.

### Patients and samples

Ten diagnostic liver cancer patients were included in this study. Primary liver cancer tissues and their corresponding tumor-free liver tissues (TFLs) were obtained from surgical resection. The clinical information of these ten diagnostic liver cancer patients was shown in Table.1. This study was approved by the medical ethical committee of the Second Hospital of Nanjing, Nanjing University of Chinese Medicine. Written informed consent was obtained from all donors in accordance with the Declaration of Helsinki (1964) before tissue sample collection.

**Table 1:** Clinical information of liver cancer patients

	HCC (*n* = 10)
Age	54.00 ± 4.32
Gender	
Male	8
Female	2
HBV	
Positive	7
Negative	3
Tumor number	
Solitary	6
Multiple	4
ES grade	
I–II	4
III–IV	6
BCLC stage	
A	6
B	2
C	2
Pathologic N	
N0	8
N1	2
Pathologic M	
M0	7
M1	3

*ES grade* Edmondson-Steiner grade, *BCLC stage* Barcelona Clinic Liver Cancer stage

### Immunohistochemistry

Paraffin-embedded liver cancer tissues were sectioned into 3-μm pieces. Tumor sections were incubated with rabbit polyclonal antibodies against LILRB1, LILRB2, LILRB4, and LILRB5 and rat polyclonal antibodies against LILRB3 at 4 °C for 12 h. The antibodies against LILRB1, LILRB3, LILRB4, and LILRB5 were from Abcam (product code: ab229186, ab271287, ab229747, ab121357) and the antibody against LILRB2 was from Invitrogen (product code: PA5-103913). To detect primary antibody binding, the sections were conjugated with goat anti-rabbit IgG H&L (HRP) (Abcam, Cambridge, UK), goat anti-rat IgG H&L (HRP polymer) (Abcam, Cambridge, UK), or goat anti-rabbit IgG (HRP) (Invitrogen, Carlsbad, CA, USA) at 25 °C for 2 h. Finally, the tumor sections were visualized with 3,3-diaminobenzidine and counterstained with hematoxylin. The tumor sections were observed by light microscopy. According to the literature, the *H*-score was used to evaluate the results [26]. In brief, the calculation method was as follows: percentage of weak staining + 2 × percentage of moderate staining + 3 × percentage of strong staining, giving a range of 0–300.

### Isolation of single cells and flow cytometry analysis

Liver tumor tissues and TFL were cut into approximately 1-mm^3^ pieces and digested with 0.125 mg/ml collagenase IV (Sigma-Aldrich, St. Louis, MO) and 0.2 mg/ml DNAse I (Roche, Indianapolis, IN) in Hanks solution for 1 h. Then, cells were isolated by filtration of liver slurry through 40-μm filters (BD Biosciences, San Jose, CA, USA), followed by centrifugation at 400 *g* for 10 min. These cells were washed twice with PBS (pH = 7.4) with 1% FBS (Invitrogen, Carlsbad, CA, USA). Next, cells were labeled with monoclonal antibodies, including CD3-PerCp (OKT3), CD56-PeCy7 (MEM-188), CD16-APC-Cy7 (CB16), and LIVE/DEAD® Aqua, for 30 min at 4 °C (Thermo Fisher Scientific, Waltham, MA, USA). After washing twice in PBS with 1% FBS, cells were analyzed by a FACS Canto II flow cytometer (BD Biosciences, San Jose, CA, USA).

### Statistical analysis

The percentage of NK cell subsets are expressed as the mean ± SEM. Student’s 𝑡-test was used to analyze the results of immunohistochemistry and the difference of NK cell subsets between tumor and their corresponding TFL. Significance was defined as < 0.05.

## Results

### Transcriptional levels of the LILRB family in patients with liver cancer

**Fig. 1:**
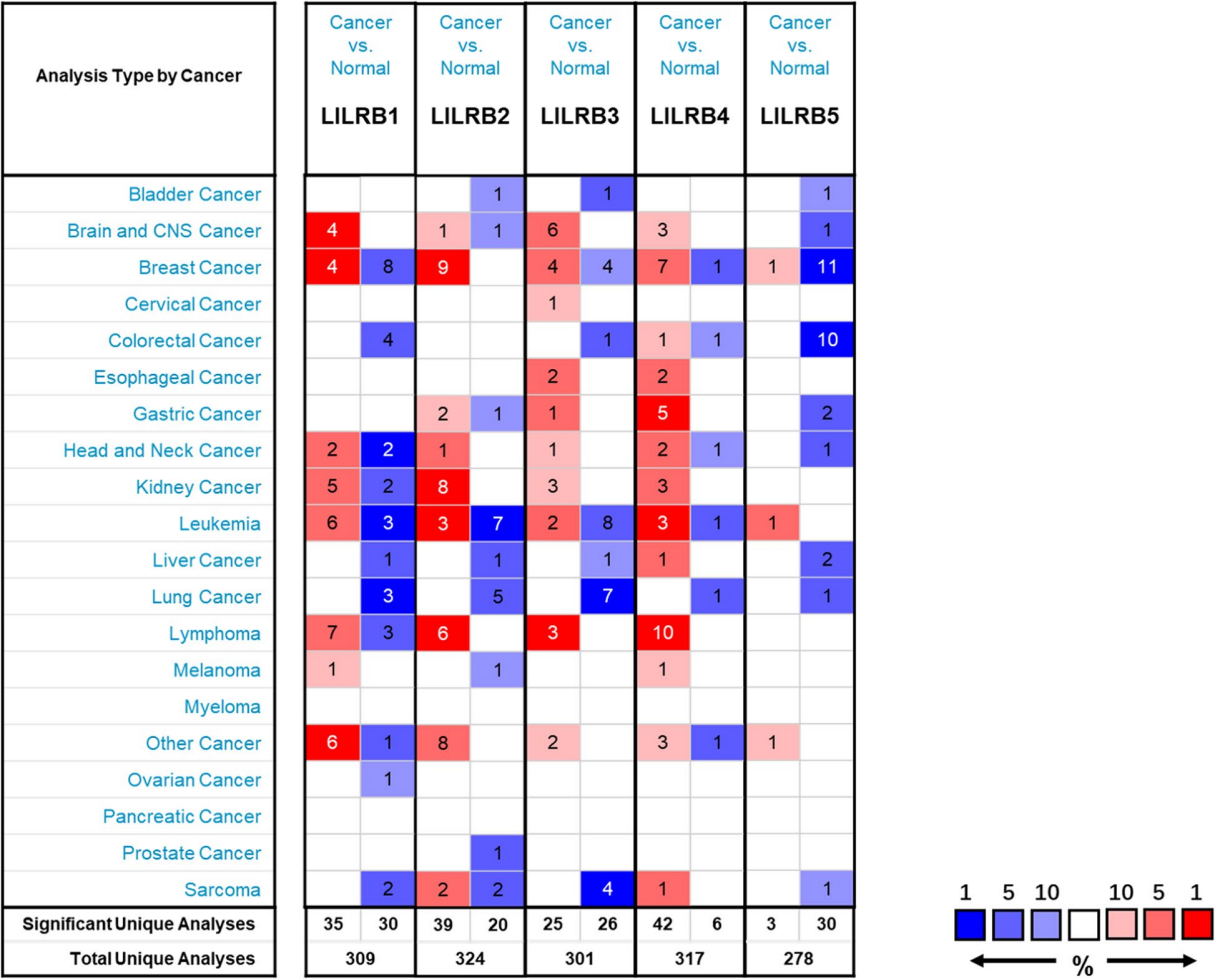
The transcription levels of the LILRB family in different types of human cancers. The figure was generated from the Oncomine database under the conditions of *p* < 0.05, fold change > 1.5, gene bank: top 10%, data type: mRNA. The cell number represented the dataset numbers that meet the conditions. The cell color was determined by the best gene rank percentile for the analyses within the cell. Blue represents underexpression and red represents overexpression

The LILRB family contains five subtypes: LILRB1, LILRB2, LILRB3, LILRB4, and LILRB5. We compared the mRNA expression of the LILRB family in different cancers with that in corresponding normal tissues based on the Oncomine database. As shown in Fig. 1, compared with that of normal tissues, in addition to LILRB4, other LILRB family members were downregulated in liver tumor tissues. All the data came from four datasets, and all the tumor data came from HCC [27–30]. Three of the datasets had the same results even if two datasets did not achieve the condition of fold change > 1.5, while Mas’s dataset had the opposite results (Table 2).

**Table 2:** Datasets of the LILRB family mRNA expression in Liver cancer (Oncomine database)

Gene	Type of liver cancer versus normal liver tissue	Fold change^a^	*p* value^b^	*t* test^c^	Source and/or reference
LILRB1	Hepatocellular carcinoma	− 1.602	0.001	− 3.569	Wurmbach et al. [22]
	Hepatocellular carcinoma	− 1.251	7.77E−22	− 10.05	Roessler et al. [23]
	Hepatocellular carcinoma	− 1.182	6.40E−04	− 3.466	Roessler et al. [23]
	Hepatocellular carcinoma	1.211	1.000	3.939	Mas et al. [24]
LILRB2	Hepatocellular carcinoma	− 2.098	1.32E−12	− 7.529	Chen et al. [25]
	Hepatocellular carcinoma	− 1.441	3.20E−30	− 12.256	Roessler et al. [23]
	Hepatocellular carcinoma	− 1.281	1.20E−04	− 4.027	Roessler et al. [23]
	Hepatocellular carcinoma	− 1.708	0.004	− 3.093	Wurmbach et al. [22]
	Hepatocellular carcinoma	1.068	0.851	1.058	Mas et al. [24]
LILRB3	Hepatocellular carcinoma	− 2.078	7.05E−10	− 6.419	Chen et al. [25]
	Hepatocellular carcinoma	− 1.391	3.58E−33	− 12.99	Roessler et al. [23]
	Hepatocellular carcinoma	− 1.341	0.002	− 3.056	Roessler et al. [23]
	Hepatocellular carcinoma	− 1.322	0.008	− 2.79	Wurmbach et al. [22]
	Hepatocellular carcinoma	1.108	0.971	1.940	Mas et al. [24]
LILRB4	Hepatocellular carcinoma	1.7	8.96E−11	8.127	Mas et al. [24]
	Hepatocellular carcinoma	− 1.011	0.644	− 0.372	Roessler et al. [23]
	Hepatocellular carcinoma	− 1.163	0.836	− 1.005	Wurmbach et al. [22]
	Hepatocellular carcinoma	− 1.101	1.000	− 4.829	Roessler et al. [23]
LILRB5	Hepatocellular carcinoma	− 1.656	2.38E−63	− 20.022	Roessler et al. [23]
	Hepatocellular carcinoma	− 2.826	9.09E−05	− 5.016	Wurmbach et al. [22]
	Hepatocellular carcinoma	− 1.321	6.53E−07	− 5.702	Roessler et al. [23]
	Hepatocellular carcinoma	1.023	0.658	0.410	Mas et al. [24]

^a^Fold change was calculated as a binary logarithm of the mRNA expression between liver cancer and normal liver tissue. A positive value means the mRNA expression of LILRB in liver cancers was larger than that in normal liver tissue. A negative value means the opposite

^b^*p* values were calculated by Student’s *t* test

^c^The t values after Student’s *t* test

**Fig. 2:**
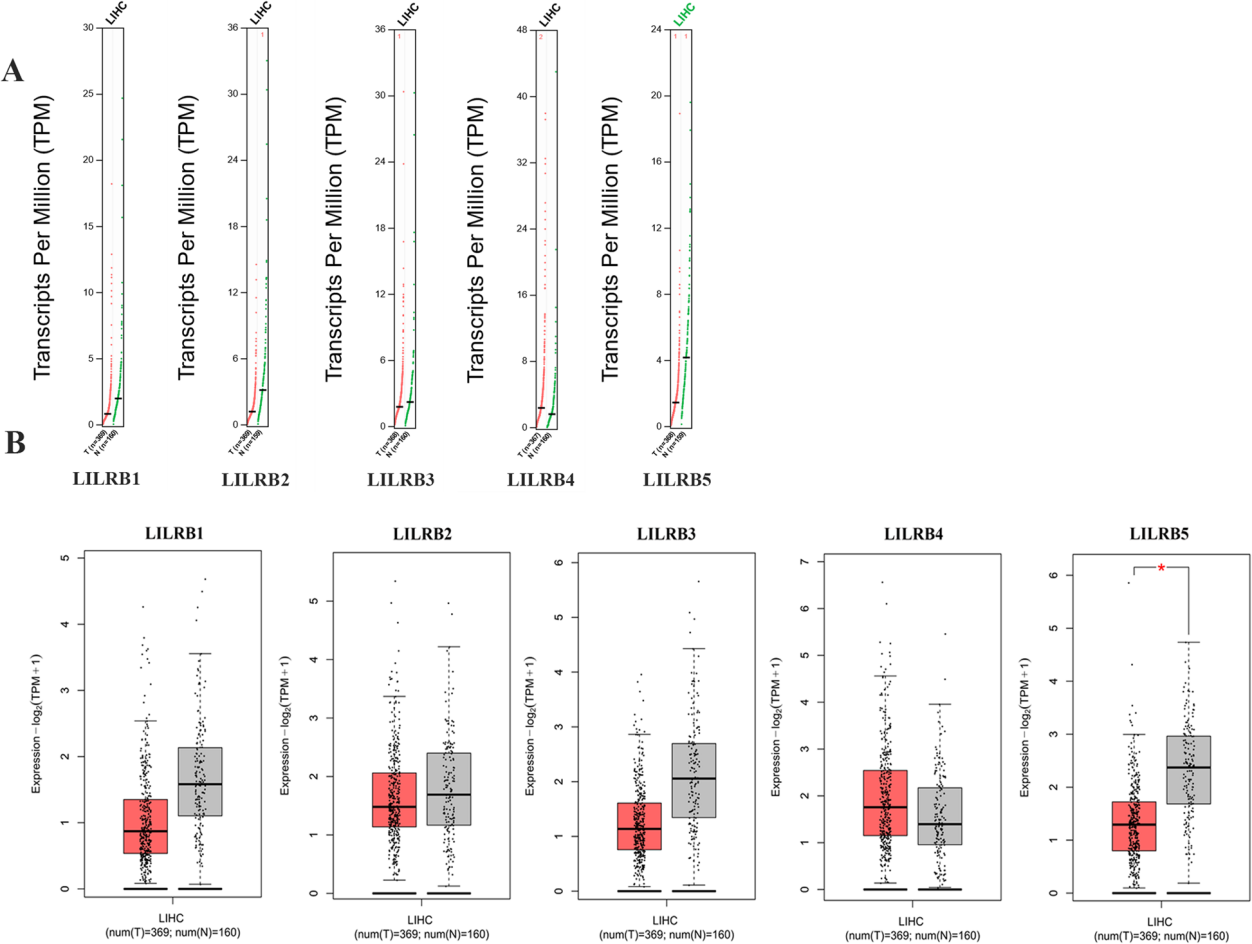
The transcription expression levels of the LILRB family in liver cancer (GEPIA 2.0). **A** Scatter diagram of individual LILRB member expression. T means tumor tissue and N means normal tissue. The number of HCC tumor tissues and normal tissues was noted in the figure. **B** Box plot of individual LILRB member expression. The red color represented tumor tissues, and the gray color represented normal tissues. The number of HCC tumor samples was 369 and the number of normal tissues was 160. This figure was generated under the conditions of log_2_(fold change) cutoff: 1, *q*-value cutoff: 0.01. The ANOVA was used for tumor vs paired normal samples. *p < 0.05

To verify the above results further, GEPIA 2.0 was used to compare the mRNA expression of the LILRB family between liver tumor tissues and normal tissues. The LIMMA method was used to compare liver tumor tissues and their paired normal samples regarding mRNA expression of the LILRB family. The results showed that LILRB1, LILRB2, LILRB3, and LILRB5 were downregulated, and compared with liver normal tissues, LILRB4 expression was upregulated in liver tumor tissues, which was consistent with the results in the Oncomine database (Fig. 2).

**Fig. 3:**
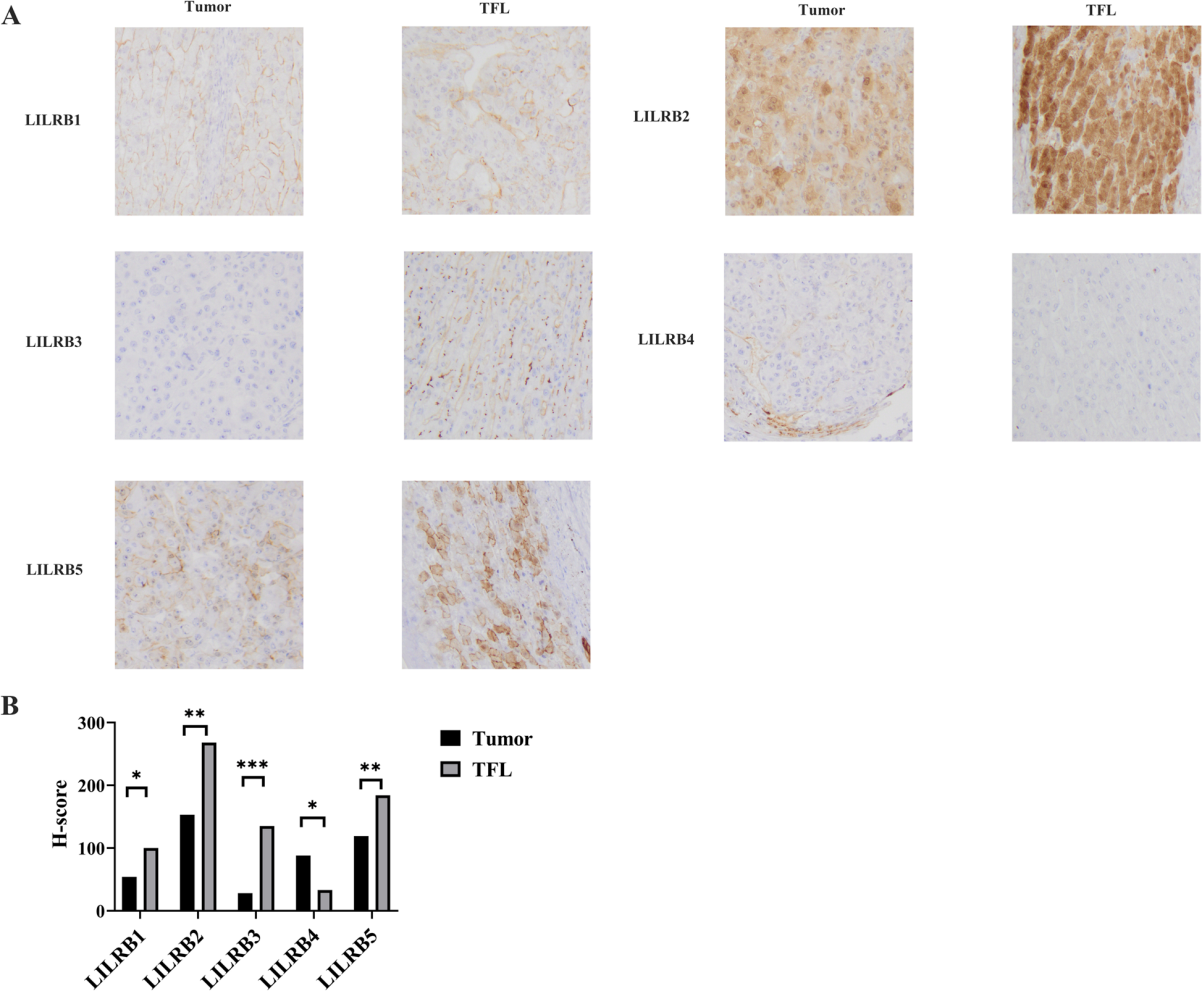
The expression of LILRB family members in liver cancer by immunohistochemical analysis. **A** Representative IHC images of LILRB family members’ expression in the microarray that contained 10 cases of liver tumor tissues and their TFL. Multiple images were taken and a representative one was presented. Brown granules were defined as positive staining of LILRB family members. The magnification was 100×. **B** The histogram of *H*-score about each LILRB family members. Student’s *t* test was used for tumor vs TFL samples. *p < 0.05, **p<0.01, ***p<0.001

To assess LILRB expression at the protein level, we detected LILRB family expression in liver tumors and their corresponding TFLs by using immunohistochemistry. We found that the expression of LILRB1, LILRB2, LILRB3 and LILRB5 was lower in liver tumors than in TFL, while the expression of LILRB4 was high in liver tumors than in TFL (Fig. 3).

### The relationship between the LILRB family and tumor stage in liver cancer patients

**Fig. 4:**
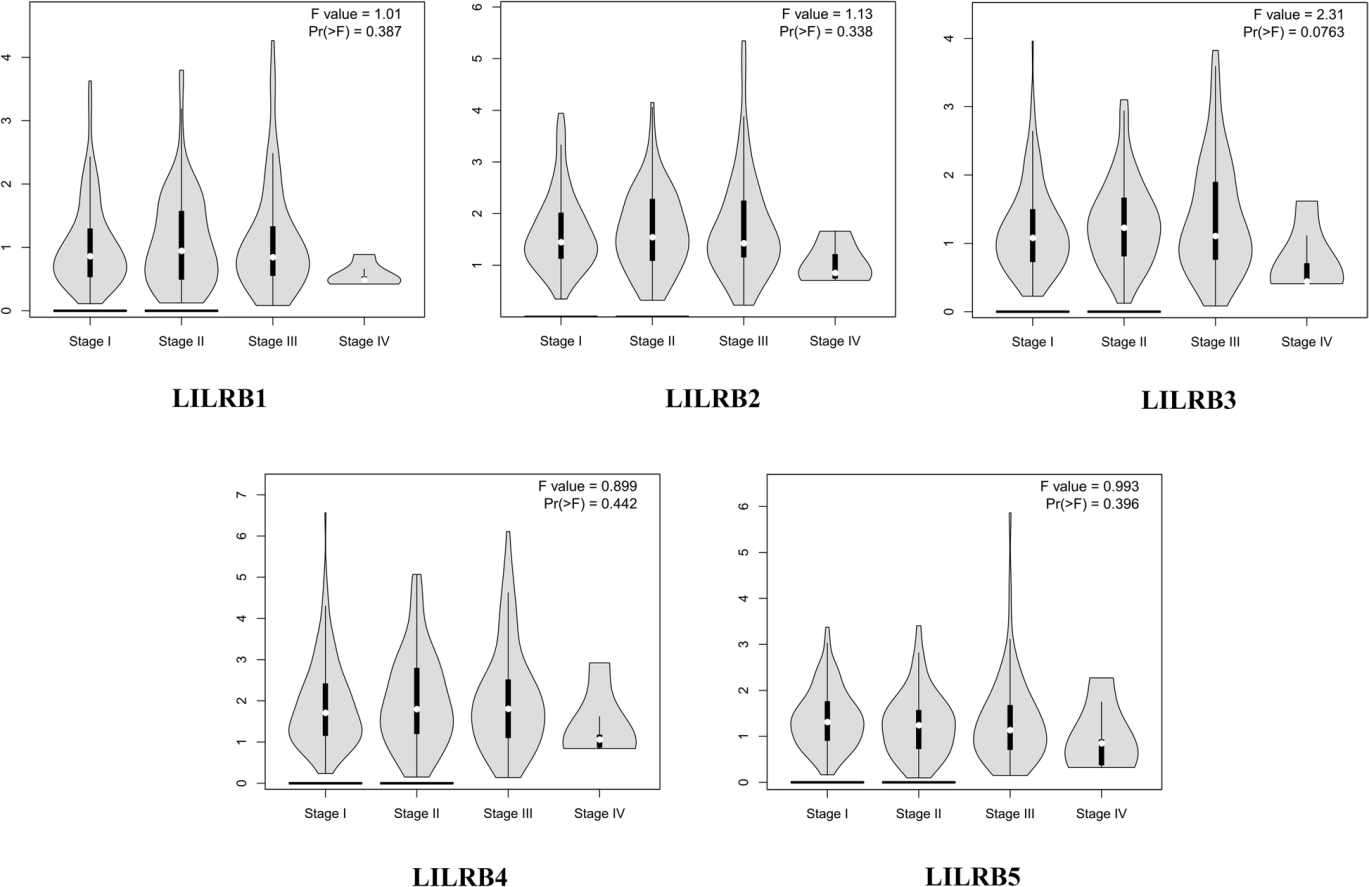
The correlation between LILRB family expression and tumor stages in liver cancer patients (GEPIA 2.0). Patients with liver cancer were divided into four groups based on disease stage, including stages I, II, III, and IV. The correlation between LILRB family members’ expression and tumor stages was analyzed by one-way ANOVA.

We compared the relationship between LILRB family expression and four tumor stages in liver cancer patients by using GEPIA 2.0. One-way ANOVA was used to analyze these results. In agreement with the literature [16], although there was no significant correlation between LILRB family expression and tumor stage in liver cancer patients, compared with that in other stages of liver cancer patients, the expression of the LILRB family in stage IV liver cancer patients decreased (Fig. 4).

### Prognostic value of LILRB family mRNA expression in all liver cancers

**Fig. 5:**
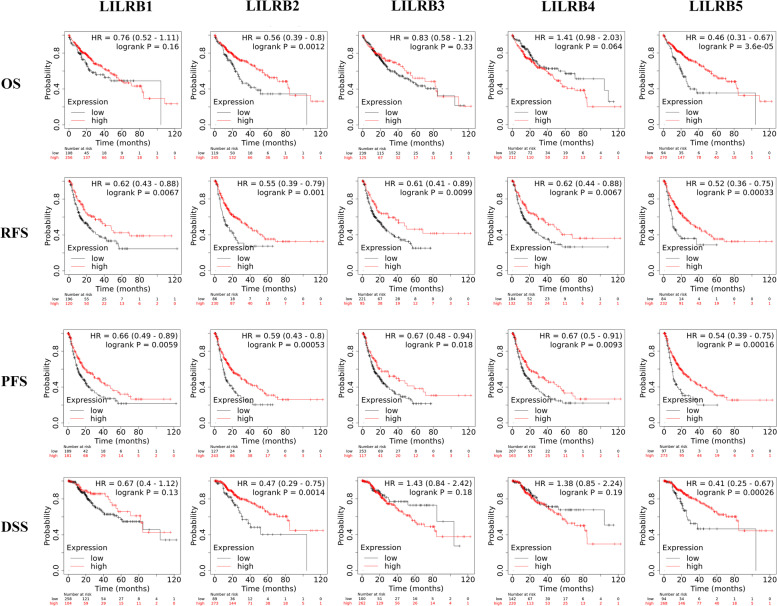
Comparison of Kaplan–Meier survival curves of LILRB family members’ low expression and high expression in liver cancers (Kaplan–Meier Plotter). The expression of LILRB family members related to OS(*n* = 364), RFS(*n* = 316), PFS(*n* = 370) and DSS(n=362) in liver cancers. Transcriptional expression levels of the LILRB family and patient survival information were obtained from GEO, EGA, and TCGA. The best cutoff values were auto-selected by the tool of Kaplan–Meier Plotter

Kaplan–Meier Plotter was used to examine the prognostic value of LILRB family mRNA expression levels in all liver cancers. We compared the correlation between mRNA expression of LILRB family members and OS, RFS, PFS, and DSS of liver cancer patients. The results revealed that patients in LILRB2- and LILRB5-low groups had shortened OS and DSS and low expression of all LILRB family members predicted poorer patients’ RFS and PFS (*p* < 0.05) (Fig. 5). Median survival times of LILRB-high groups in OS, PFS, PFS, and DSS were longer than those of LILRB-low groups, when there was a statistical difference between these two groups (Table 3).

**Table 3:** Median survival time of LILRB low- and high-expression liver patients in OS, RFS, PFS, and DSS (Kaplan–Meier plots)

Survival outcome	Expression (month)^a^	LILRB1	LILRB2	LILRB3	LILRB4	LILRB5
OS	Low-expression cohort	46.60	30.00	54.10	104.20	25.60
	High-expression cohort	56.50	71.00	70.50	49.70	70.50
RFS	Low-expression cohort	21.87	14.33	21.93	21.30	10.50
	High-expression cohort	47.73	37.23	47.73	47.73	34.40
PFS	Low-expression cohort	16.73	12.80	19.53	16.83	10.50
	High-expression cohort	30.40	30.10	37.67	36.10	29.30
DSS	Low-expression cohort	84.73	40.33	104.17	31.03	37.83
	High-expression cohort	84.40	84.73	70.53	33.50	84.73

^a^The cutoff values distinguished by low expression and high expression were computed by the Kaplan–Meier Plotter

Next, we further investigated the prognostic value of LILRB family mRNA expression in liver cancer with different histologic stages by using Kaplan–Meier Plotter. The results were as follows: (1) The high mRNA expression of LILRB1 was associated with better RFS and PFS in stages I and II. (2) The high mRNA expression of LILRB2 was associated with longer OS, RFS and PFS in stages I, II, and III. High mRNA expression of LILRB2 was predicted to have better DSS in stages II and III. (3) High expression of LILRB3 mRNA was correlated with better RFS and PFS in stage II, while low expression of LILRB3 mRNA was associated with better DSS in stage I. (4) The low mRNA expression of LILRB4 was associated with better DSS in stage I. However, the high expression of LILRB4 mRNA was correlated with better RFS and PFS in stage II as well as better PFS in stage III. (5) High mRNA expression of LILRB5 was predicted to be associated with better OS in stage II and III patients and with better PFS in stage II patients, while low mRNA expression of LILRB5 was associated with better OS in stage I patients (Supplementary Table S1).

### The relationship between LILRB family expression and immune cell infiltration

**Fig. 6:**
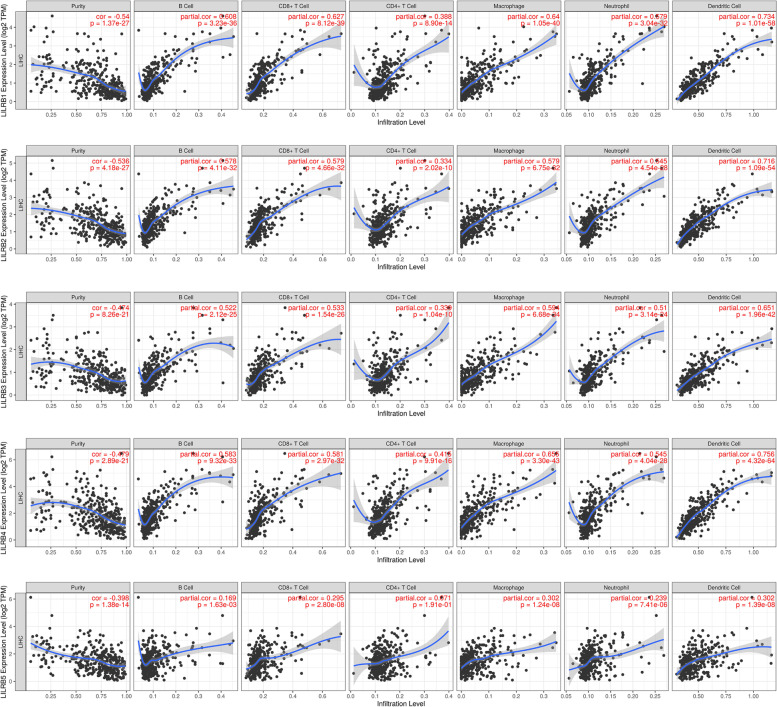
Correlation of LILRB family expression with immune infiltration level in liver cancer (TIMER). LILRB family members’ expression in HCC tissues negatively correlated with tumor purity and positively correlated with infiltration levels of B cells, CD8^+^ T cells, CD4^+^ T cells, macrophages, neutrophils, and dendritic cells (*n* = 371). The correlations were analyzed using Spearman’s correlation measure. LIHC, liver hepatocellular carcinoma

The LILRB family is considered to be an immune inhibitory receptor. TILs are associated with prognostic indicators for liver cancer [31]. Therefore, we speculated that there was a positive correlation between LILRB family expression and TIL infiltration in liver cancer patients. TIMER database was used to analyze the suspected association. The results showed that all LILRB family members were significantly associated with tumor purity, B cells, CD8^+^ T cells, CD4^+^ T cells, macrophages, neutrophils, and dendritic cells (Fig. 6).

The TISIDB database was used to assess the relationship between LILRB family expression and TIL subsets. Although there was no significant correlation of LILRB5 expression with activated CD4^+^ T cells and CD56^dim^ natural killer cells, the expression of other LILRB family members in liver cancer was strongly associated with various TIL subsets (Supplementary Tables S2, S3, S4, S5, S6). Together these findings suggested that the LILRB family plays an important role in the recruitment and regulation of immune infiltrating cells in liver cancer.

### Predicted functions and pathways of the LILRB family in liver cancer

**Fig. 7:**
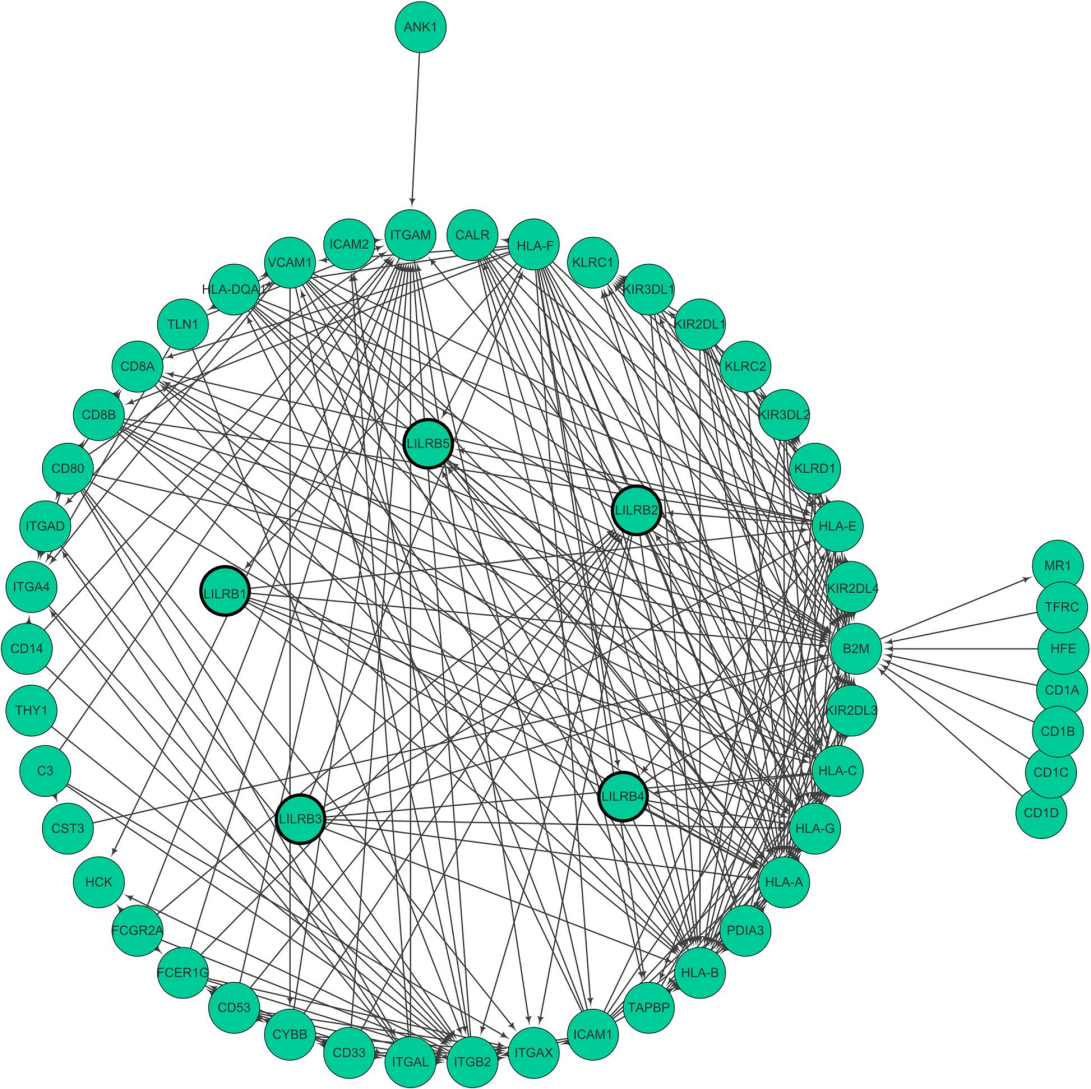
Analysis of LILRB family-related proteins based on a protein-protein interaction network. Using the STRING database, a total of 50 proteins were filtered into the protein PPI network. Nodes represent interacting proteins, and the 452 edges represent known or predicted interactions

To understand the biological significance and consequences of the protein profiling of the LILRB family, we constructed a PPI network by using STRING. The Cytoscape software was used to process it. As shown in Fig. 7, PPI analysis showed known and predicted interactions between LILRB family members and 50 proteins.

**Fig. 8:**
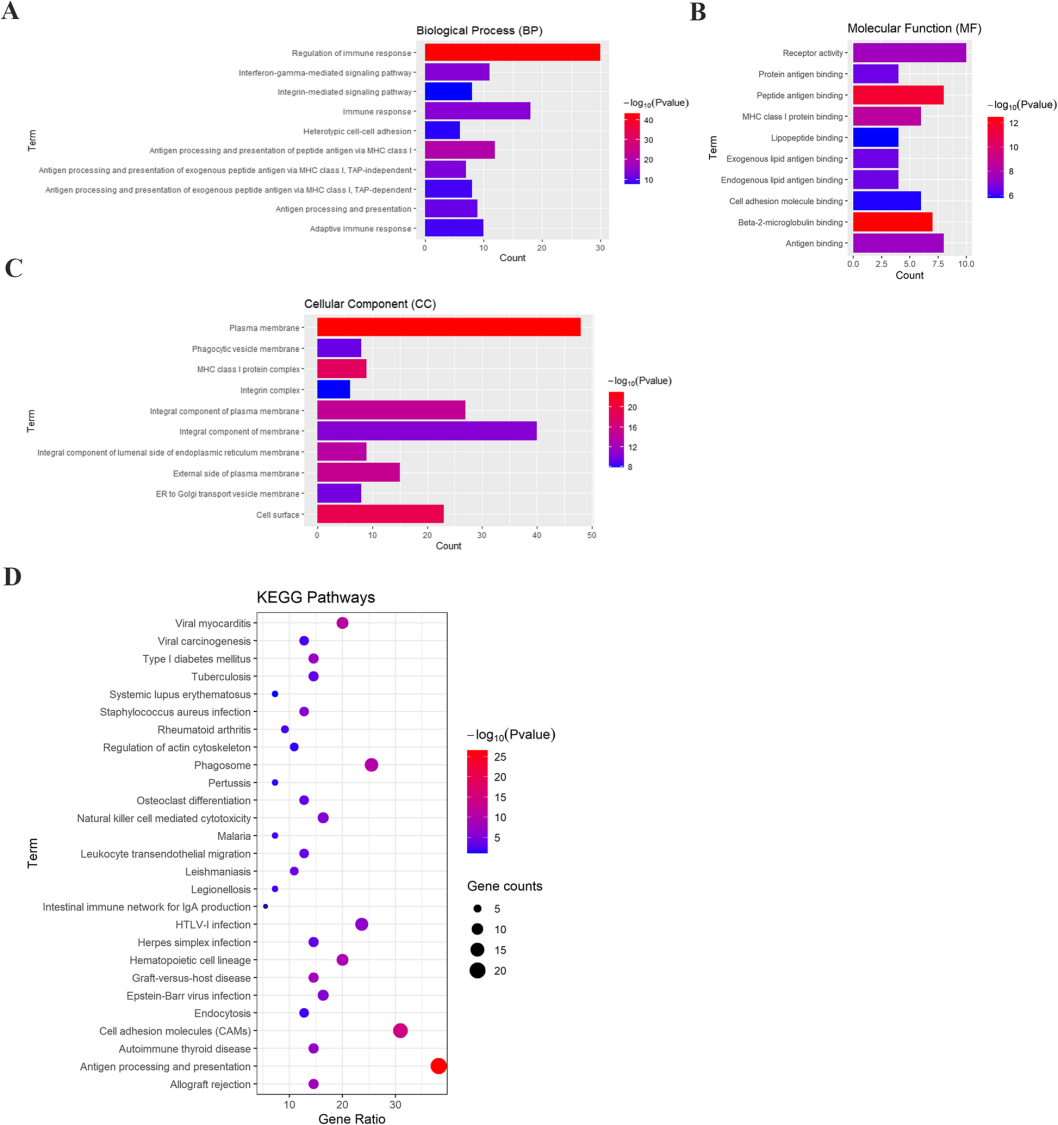
GO and KEGG analyses of the LILRB family and their related genes. The DAVID online database was used to perform the GO and KEGG analyses. **A**–**C** The top 10 items of the GO analysis for BP, CC, and MF of the LILRB family and their related genes, respectively. **D** The KEGG pathways of the LILRB family and their related genes

Next, GO and KEGG analyses for the LILRB family and these 50 genes were performed using the DAVID online tool. The GO analysis showed that there were 38 items of biological process (BP), 25 items of cellular component (CC), and 23 items of molecular function (MF) with FDR less than 0.05. The first 10 items were used to plot. The GO analysis for BP showed that LILRB family members and their related genes were mostly enriched in the immune response and antigen processing and presentation of antigens (Fig. 8A). The GO analysis for MF showed that LILRB family members and their related genes were associated with beta-2-microglobulin binding, peptide antigen binding, and MHC class I protein binding (Fig. 8B). The GO analysis for CC revealed that LILRB family members and their related genes were significantly enriched in the plasma membrane and cell surface (Fig. 8C). In KEGG analysis, 27 pathways were related to the function of LILRB family members (Fig. 8D). Among them, antigen processing and presentation, cell adhesion molecules (CAMs), phagosomes, natural killer cell-mediated cytotoxicity, and leukocyte transendothelial migration were involved in antitumor immunity in liver cancer.

Based on the KEGG results and previous researches [32–34], we hypothesized that LILRB family members and their related genes are involved in the pathways of antigen processing and presentation and natural killer (NK) cell-mediated cytotoxicity.

**Fig. 9:**
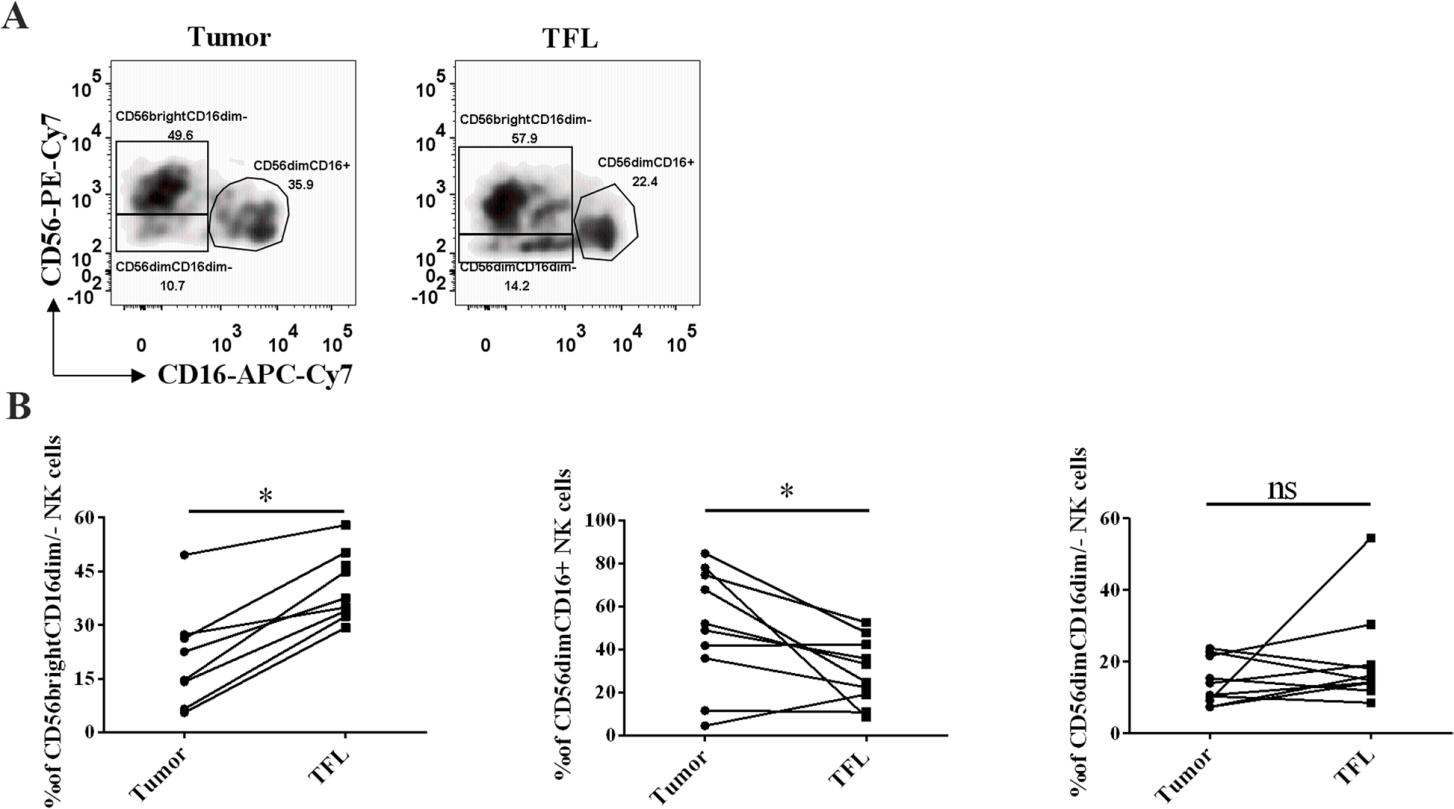
The expression of NK cell subsets in liver cancer. **A** Flow cytometry was used to detect NK cells. NK cell subsets were identified by three unique CD56 and CD16 expression patterns in the gate of Live^+^CD3^-^CD56^bright/dim^. **B** The percentage of NK cell subsets from liver tumors and the corresponding TFL (*n* = 10). *p < 0.05

Our previous study verified that compared with their corresponding tumor-free liver tissues (TFLs), the percentage of infiltrating CD1c^+^ myeloid DCs (mDCs) was significantly decreased in liver tumor tissues [7]. Considering that infiltrating NK cells in liver cancer might be involved in the signaling of the LILRB family, we further compared the percentage of NK cells in liver tumor tissues and the corresponding TFL. Human NK cells can be segregated into three major subsets: CD56^bright^CD16^dim/−^, CD56^dim^CD16^+^, CD56^dim^CD16^dim/−^ NK cells. The strategy of gating these three NK cells subsets were according to the literature [35]. The results shown that the percentage of CD56^bright^CD^dim/−^ NK cell in liver tumor tissues was lower than that from TFL tissues (Tumor vs TFL: 30.84 % ± 7.77 % vs 43.85 % ± 4.12 %, *p* < 0.05) (Fig. 9). In contrast, the percentage of CD56^dim^CD16^+^ NK cell in liver tumor tissues was higher than that from TFL tissues (Tumor vs TFL: 49.98 % ± 8.66 % vs 29.73 % ± 4.78 %, *p* < 0.05).

## Discussion

The LILRB family is considered to be an immune inhibitory receptor. Most studies have focused on the expression of the LILRB family on immune cells in many cancers [19, 36]. A recent study showed that LILRB2 was expressed in lung cancer tissues and that its expression resulted in poor patient OS [37]. On the contrary, Cheng et al. [16] found that LILRB1 in hepatocarcinoma cells might integrate with SHP1 to exert an antitumor effect in liver cancer patients. Hence, the expression of LILRB family members have observed in tumor cells with no clear function. Moreover, the expression of all LILRB family members and their relationships with prognosis in liver cancer patients have yet to be determined. In this study, a series of bioinformatics analysis methods and experiments were used to explode the expression, prognosis and potential function of the LILRB family in liver cancer.

First, we assessed the expression of the LILRB family in liver tumor tissues and its correlation with the survival of liver cancer patients. The Oncomine and GEIPA 2.0 databases confirmed that LILRB1, LILRB2, LILRB3, and LILRB5 expression was lower in liver tumor tissues than in normal tissues and that LILRB4 was overexpressed in liver tumor tissues. To further verify these results, we assessed LILRB family member expression at the protein level by using immunohistochemistry. The results were in accordance with the mRNA expression of the LILRB family, which was also consistent with a previous study [16]. In contrast, some studies proved that LILRB2 was expressed at higher levels in HCC tissues, which needs further verification with large samples in future studies [37, 38]. In addition, we also used Kaplan–Meier Plotter to explode the prognostic merit of LILRB family member expression in liver cancer. Our results showed that LILRB2 and LILRB5 expression was positively associated with OS and DSS and that the mRNA expression of all LILRB family members was significantly positively correlated with RFS and PFS in liver cancer patients. Hence, these results robustly indicated that the LILRB family was a potential prognostic biomarker in liver cancer patients.

Numerous studies have proven that the LILRB family plays an immunosuppressive role in the immune system. These immune inhibitory receptors could restrict T cell infiltration and killing ability, leading to impaired antitumor responses [39]. Scientists have discovered that PIR-B is expressed on murine B cells and myeloid cells, as the ortholog for human LILRB1/B2, blocked the access of CD8αα to MHC-I, resulting in weaker antitumor immunity [40]. Researchers have also found that disruption of either MHC class I or LILRB1 enhanced phagocytosis of tumor cells in macrophages [36]. For these reasons, we compared the correlation between the LILRB family and infiltrating immune cells. The mRNA expression of the LILRB family was inversely proportional to the purity of the liver tumor, while LILRB family expression was proportional to immune cells, including B cells, CD8^+^ T cells, CD4^+^ T cells, macrophages, neutrophils and dendritic cells. The positive correlation between LILRB family members’ expression and immune cells implicated the role of LILRBs in recruiting and regulating tumor immunology in liver cancer.

Previous studies have explored some mechanisms of the LILRB family expressed on immune cells in the immune system. Khanolkar et al. found that LILRB1 on dendritic cells increased the expression of the NF-κB regulator ABIN1/TNIP1, resulting in suppression of the stimulatory effect of dendritic cells on T cells. Research has also discovered that LILRB2 expressed on tumor-associated myeloid cells inhibited the activation of SHP1/2, AKT and STAT6, leading to restraint of the function of M1-like macrophages and promotion of the function of M2-like macrophages [41]. However, these mechanisms are concerned with the expression of the LILRB family on immune cells, and the mechanisms of the LILRB family expressed in the tumor microenvironment, especially on solid tumors, have not yet been discovered clearly. We identified 50 potential and validated proteins that interacted with the LILRB family by using STRING. The KEGG analysis results and previous experiments showed that the LILRB family might be involved in antigen processing and presentation and NK cell pathways [42]. NK cells have been studied as three separate populations: CD56^bright^CD16^dim/−^, CD56^dim^CD16^+^, CD56^dim^CD16^dim/−^ NK cells. CD56^bright^CD16^dim/−^ NK cells secreted cytokines to kill tumor cells and regulate the immune system. CD56^dim^CD16^+^ NK cells primarily demonstrated cytotoxicity effect. CD56^dim^CD16^dim/−^ NK cells might be the immediate precursors of the CD56^dim^CD16^+^ subset, which remained to be clarified in the future [43]. Consistent with the literature, CD56^dim^CD16^+^ NK cells were significantly reduced in liver tumor tissues compared with TFL, resulting in secreting less cytokines that killed tumor cells [44]. This might due to the reduced expressed LILRB family members on tumor cells, leading to recruiting less NK cells.

## Conclusions

In this study, using bioinformatics analysis, we identified decreases in LILRB1, LILRB2, LILRB3, and LILRB5 and increases in LILRB4 in liver tumor tissues, which were confirmed at the mRNA and protein levels. Moreover, survival analysis revealed that LILRB2 and LILRB5 mRNA levels were significantly positively associated with OS and DSS and that the mRNA expression of all LILRB family members was significantly positively correlated with RFS and PFS. High expression of LILRB family members was associated with increased infiltration of immune cells, including B cells, CD8^+^ T cells, CD4^+^ T cells, macrophages, neutrophils, and dendritic cells. Finally, PPI prediction analysis of the LILRB family and KEGG analysis suggested that LILRB family members might be involved in antigen processing and presentation and natural killer cell pathways during the development of liver cancer.

## Supplementary Information

**Additional file 1.**

## Data Availability

The datasets used and/or analyzed during the current study are available from the corresponding author on reasonable request. All the databases used in this study were below: Oncomine database: www.oncomine.org GEPIA database: http://gepia2.cancer-pku.cn/#index Kaplan–Meier Plotter: http://kmplot.com/analysis/index.php?p=service&cancer=liver_rnaseq Timer: https://cistrome.shinyapps.io/timer/ TISIDB: http://cis.hku.hk/TISIDB/ STRING database: https://string-db.org/cgi/input.pl DAVID database: https://david.ncifcrf.gov/summary.jsp

## Abbreviations

LILRB: Leukocyte immunoglobulin-like receptor subfamily B
HCC: Hepatocellular carcinoma
ICC: Intrahepatic cholangiocarcinoma
HBV: Hepatitis B virus
HCV: Hepatitis C virus
AFP: Alpha-fetoprotein
GEPIA: Gene expression profiling interactive analysis
OS: Overall survival
RFS: Relapse-free survival
PFS: Progression-free survival
DSS: Disease-free survival
Timer: Tumor immune estimation resource
TILs: Tumor-infiltrating lymphocytes
PPI: Protein-protein interactions
GO: Gene ontology
KEGG: Kyoto Encyclopedia of Genes and Genomes
CAMs: Cell adhesion molecules
APCs: Antigen-presenting cells
NK cells: Natural killer cells
TFL: Tumor-free liver tissues
