# Supplementary material for: Analysis of the expression and prognosis for leukocyte immunoglobulin-like receptor subfamily B in human liver cancer
Source: World J Surg Oncol. 2022 Mar 24;20:92. doi: 10.1186/s12957-022-02562-w (PMC8943947; doi:10.1186/s12957-022-02562-w)
Supplement: Supplementary file 1 — Additional file 1. [file 12957_2022_2562_MOESM1_ESM.docx]

**Supplementary Table.1 Survival analyses of the LILRB family with different stages in liver cancer (Kaplan-Meier Plotter)**

|  | Survival | Stage I | | | Stage II | | | Stage III | | | Stage IV | | |
| --- | --- | --- | --- | --- | --- | --- | --- | --- | --- | --- | --- | --- | --- |
| Gene | outcome | No.^a^ | HR | P-value | No. | HR | P-value | No. | HR | P-value | No. | HR | P-value |
|  |  |  | (95 %CI） |  |  | (95 %CI） |  |  | (95 %CI） |  |  | (95 %CI） |  |
| LILRB1 | OS | 170 | 0.74 | 0.39 | 83 | 0.6 | 0.23 | 83 | 0.6 | 0.096 | 4 | - | - |
|  |  |  | (0.38-1.47) |  |  | (0.26-1.39) |  |  | (0.32-1.1) |  |  |  |  |
|  | RFS | 153 | 0.58 | 0.07 | 74 | 0.48 | **0.032** | 68 | 0.46 | **0.024** | 0 | - | - |
|  |  |  | (0.32-1.05) |  |  | (0.24-0.95) |  |  | (0.23-0.92) |  |  |  |  |
|  | PFS | 170 | 0.61 | 0.072 | 84 | 0.49 | **0.02** | 83 | 0.45 | **0.0067** | 5 | - | - |
|  |  |  | (0.35-1.05) |  |  | (0.27-0.9) |  |  | (0.25-0.81) |  |  |  |  |
|  | DSS | 167 | 1.77 | 0.44 | 82 | 0.38 | 0.19 | 81 | 0.61 | 0.19 | 3 | - | - |
|  |  |  | (0.41-7.73) |  |  | (0.08-1.71) |  |  | (0.29-1.28) |  |  |  |  |
| LILRB2 | OS | 170 | 0.53 | **0.047** | 83 | 0.41 | **0.03** | 83 | 0.51 | **0.027** | 4 | - | - |
|  |  |  | (0.28-1） |  |  | (0.18-0.94) |  |  | (0.27-0.94) |  |  |  |  |
|  | RFS | 153 | 0.52 | **0.023** | 74 | 0.29 | **0.0044** | 68 | 0.47 | **0.018** | 0 | - | - |
|  |  |  | (0.29-0.92) |  |  | (0.12-0.71) |  |  | (0.25-0.89) |  |  |  |  |
|  | PFS | 170 | 0.6 | **0.042** | 84 | 0.3 | **0.0028** | 83 | 0.5 | **0.014** | 5 | - | - |
|  |  |  | (0.36-0.99) |  |  | (0.13-0.69) |  |  | (0.28-0.88) |  |  |  |  |
|  | DSS | 167 | 0.45 | 0.094 | 82 | 0.23 | **0.037** | 81 | 0.42 | **0.018** | 3 | - | - |
|  |  |  | (0.18-1.17) |  |  | (0.05-1.03) |  |  | (0.2-0.88) |  |  |  |  |
| LILRB3 | OS | 170 | 2.16 | 0.058 | 83 | 1.43 | 0.36 | 83 | 0.77 | 0.44 | 4 | - | - |
|  |  |  | (0.95-4.87) |  |  | (0.66-3.1) |  |  | (0.39-1.5) |  |  |  |  |
|  | RFS | 153 | 0.73 | 0.27 | 74 | 0.28 | **0.0034** | 68 | 0.65 | 0.16 | 0 | - | - |
|  |  |  | (0.42-1.27) |  |  | (0.12-0.69) |  |  | (0.36-1.2) |  |  |  |  |
|  | PFS | 170 | 1.24 | 0.44 | 84 | 0.39 | **0.01** | 83 | 0.64 | 0.11 | 5 | - | - |
|  |  |  | (0.72-2.13) |  |  | (0.19-0.82) |  |  | (0.36-1.12) |  |  |  |  |
|  | DSS | 167 | 5.18 | **0.014** | 82 | 0.51 | 0.3 | 81 | 0.55 | 0.1 | 3 | - | - |
|  |  |  | (1.2-22.35) |  |  | (0.14-1.88) |  |  | (0.27-1.14) |  |  |  |  |
| LILRB4 | OS | 170 | 1.44 | 0.25 | 83 | 1.49 | 0.37 | 83 | 1.57 | 0.13 | 4 | - | - |
|  |  |  | (0.77-2.69) |  |  | (0.62-3.59) |  |  | (0.87-2.82) |  |  |  |  |
|  | RFS | 153 | 0.64 | 0.12 | 74 | 0.34 | **0.019** | 68 | 0.57 | 0.12 | 0 | - | - |
|  |  |  | (0.36-1.12) |  |  | (0.13-0.88) |  |  | (0.28-1.17) |  |  |  |  |
|  | PFS | 170 | 0.76 | 0.28 | 84 | 0.36 | **0.017** | 83 | 0.53 | **0.033** | 5 | - | - |
|  |  |  | (0.46-1.26) |  |  | (0.15-0.86) |  |  | (0.29-0.96) |  |  |  |  |
|  | DSS | 167 | 3.25 | **0.047** | 82 | 0.41 | 0.13 | 81 | 0.74 | 0.42 | 3 | - | - |
|  |  |  | (0.95-11.11) |  |  | (0.12-1.34) |  |  | (0.35-1.55) |  |  |  |  |
| LILRB5 | OS | 170 | 1.89 | **0.045** | 83 | 0.35 | **0.015** | 83 | 0.31 | **0.0025** | 4 | - | - |
|  |  |  | (1.01-3.54) |  |  | (0.14-0.84) |  |  | (0.14-0.69) |  |  |  |  |
|  | RFS | 153 | 0.62 | 0.095 | 74 | 0.5 | 0.073 | 68 | 0.56 | 0.058 | 0 | - | - |
|  |  |  | (0.35-1.09) |  |  | (0.23-1.08) |  |  | (0.3-1.03) |  |  |  |  |
|  | PFS | 170 | 0.64 | 0.085 | 84 | 0.47 | **0.022** | 83 | 0.62 | 0.088 | 5 | - | - |
|  |  |  | (0.38-1.07) |  |  | (0.24-0.91) |  |  | (0.36-1.08) |  |  |  |  |
|  | DSS | 167 | 0.49 | 0.13 | 82 | 0.38 | 0.11 | 81 | 0.37 | 0.023 | 3 | - | - |
|  |  |  | (0.19-1.26) |  |  | (0.11-1.28) |  |  | (0.15-0.9) |  |  |  |  |

^a^ number of patients with available clinical data

**Supplementary Table.2 Spearman correlation analysis between expression of LILRB1 and TILs in liver cancer (TISIDB)**

| Tumor-infiltrating lymphocytes | R value | P value |
| --- | --- | --- |
| Activated CD8+ T cells | 0.611 | ＜2.2E-16 |
| Central memory CD8+ T cells | 0.405 | ＜2.2E-16 |
| Effector memory CD8+ T cells | 0.768 | ＜2.2E-16 |
| Activated CD4+ T cells | 0.526 | ＜2.2E-16 |
| Central memory CD4+ T cells | 0.533 | ＜2.2E-16 |
| Effector memory CD4+ T cells | 0.560 | ＜2.2E-16 |
| T follicular helper cells | 0.830 | ＜2.2E-16 |
| γδT cells | 0.523 | ＜2.2E-16 |
| Type 1 T helper cells | 0.722 | ＜2.2E-16 |
| Type 17 T helper cells | 0.354 | 2.41E-12 |
| Type 2 T helper cells | 0.489 | ＜2.2E-16 |
| Regulatory T cells | 0.832 | ＜2.2E-16 |
| Activated B cells | 0.734 | ＜2.2E-16 |
| Immature B cells | 0.816 | ＜2.2E-16 |
| Memory B cells | 0.239 | 3.35E-06 |
| Natural killer cells | 0.485 | ＜2.2E-16 |
| CD56bright natural killer cells | 0.338 | 2.64E-11 |
| CD56dim natural killer cells | 0.176 | 0.000662 |
| Myeloid derived suppressor cells | 0.780 | ＜2.2E-16 |
| Natural killer T cells | 0.635 | ＜2.2E-16 |
| Activated dendritic cells | 0.654 | ＜2.2E-16 |
| Plasmacytoid dendritic cells | 0.415 | ＜2.2E-16 |
| Immature dendritic cells | 0.443 | ＜2.2E-16 |
| Macrophage | 0.773 | ＜2.2E-16 |
| Eosinophil | 0.490 | ＜2.2E-16 |
| Mast cells | 0.741 | ＜2.2E-16 |
| Monocyte | 0.358 | 1.47E-12 |
| Neutrophil | 0.459 | ＜2.2E-16 |

**Supplementary Table.3 Spearman correlation analysis between expression of LILRB2 and TILs in liver cancer (TISIDB)**

| Tumor-infiltrating lymphocytes | R value | P value |
| --- | --- | --- |
| Activated CD8+ T cells | 0.640 | ＜2.2E-16 |
| Central memory CD8+ T cells | 0.421 | ＜2.2E-16 |
| Effector memory CD8+ T cells | 0.784 | ＜2.2E-16 |
| Activated CD4+ T cells | 0.479 | ＜2.2E-16 |
| Central memory CD4+ T cells | 0.445 | ＜2.2E-16 |
| Effector memory CD4+ T cells | 0.521 | ＜2.2E-16 |
| T follicular helper cells | 0.798 | ＜2.2E-16 |
| γδT cells | 0.553 | ＜2.2E-16 |
| Type 1 T helper cells | 0.714 | ＜2.2E-16 |
| Type 17 T helper cells | 0.349 | 5.86E-12 |
| Type 2 T helper cells | 0.438 | ＜2.2E-16 |
| Regulatory T cells | 0.812 | ＜2.2E-16 |
| Activated B cells | 0.696 | ＜2.2E-16 |
| Immature B cells | 0.768 | ＜2.2E-16 |
| Memory B cells | 0.270 | 1.29E-07 |
| Natural killer cells | 0.510 | ＜2.2E-16 |
| CD56bright natural killer cells | 0.379 | 3.97E-14 |
| CD56dim natural killer cells | 0.231 | 6.85E-06 |
| Myeloid derived suppressor cells | 0.793 | ＜2.2E-16 |
| Natural killer T cells | 0.635 | ＜2.2E-16 |
| Activated dendritic cells | 0.640 | ＜2.2E-16 |
| Plasmacytoid dendritic cells | 0.411 | ＜2.2E-16 |
| Immature dendritic cells | 0.482 | ＜2.2E-16 |
| Macrophage | 0.772 | ＜2.2E-16 |
| Eosinophil | 0.520 | ＜2.2E-16 |
| Mast cells | 0.731 | ＜2.2E-16 |
| Monocyte | 0.372 | 1.37E-13 |
| Neutrophil | 0.460 | ＜2.2E-16 |

**Supplementary Table.4 Spearman correlation analysis between expression of LILRB3 and TILs in liver cancer (TISIDB)**

| Tumor-infiltrating lymphocytes | R value | P value |
| --- | --- | --- |
| Activated CD8+ T cells | 0.530 | ＜2.2E-16 |
| Central memory CD8+ T cells | 0.408 | ＜2.2E-16 |
| Effector memory CD8+ T cells | 0.619 | ＜2.2E-16 |
| Activated CD4+ T cells | 0.498 | ＜2.2E-16 |
| Central memory CD4+ T cells | 0.472 | ＜2.2E-16 |
| Effector memory CD4+ T cells | 0.412 | ＜2.2E-16 |
| T follicular helper cells | 0.710 | ＜2.2E-16 |
| γδT cells | 0.454 | ＜2.2E-16 |
| Type 1 T helper cells | 0.596 | ＜2.2E-16 |
| Type 17 T helper cells | 0.374 | 9.72E-14 |
| Type 2 T helper cells | 0.407 | ＜2.2E-16 |
| Regulatory T cells | 0.703 | ＜2.2E-16 |
| Activated B cells | 0.604 | ＜2.2E-16 |
| Immature B cells | 0.646 | ＜2.2E-16 |
| Memory B cells | 0.154 | 0.00293 |
| Natural killer cells | 0.433 | ＜2.2E-16 |
| CD56bright natural killer cells | 0.384 | 1.76E-14 |
| CD56dim natural killer cells | 0.279 | 4.65E-08 |
| Myeloid derived suppressor cells | 0.749 | ＜2.2E-16 |
| Natural killer T cells | 0.574 | ＜2.2E-16 |
| Activated dendritic cells | 0.599 | ＜2.2E-16 |
| Plasmacytoid dendritic cells | 0.414 | ＜2.2E-16 |
| Immature dendritic cells | 0.402 | ＜2.2E-16 |
| Macrophage | 0.727 | ＜2.2E-16 |
| Eosinophil | 0.379 | 4.16E-14 |
| Mast cells | 0.703 | ＜2.2E-16 |
| Monocyte | 0.351 | 4.09E-12 |
| Neutrophil | 0.501 | ＜2.2E-16 |

**Supplementary Table.5 Spearman correlation analysis between expression of LILRB4 and TILs in liver cancer (TISIDB)**

| Tumor-infiltrating lymphocytes | R value | P value |
| --- | --- | --- |
| Activated CD8+ T cells | 0.553 | ＜2.2E-16 |
| Central memory CD8+ T cells | 0.462 | ＜2.2E-16 |
| Effector memory CD8+ T cells | 0.682 | ＜2.2E-16 |
| Activated CD4+ T cells | 0.577 | ＜2.2E-16 |
| Central memory CD4+ T cells | 0.590 | ＜2.2E-16 |
| Effector memory CD4+ T cells | 0.508 | ＜2.2E-16 |
| T follicular helper cells | 0.785 | ＜2.2E-16 |
| γδT cells | 0.532 | ＜2.2E-16 |
| Type 1 T helper cells | 0.682 | ＜2.2E-16 |
| Type 17 T helper cells | 0.397 | 5.32E-16 |
| Type 2 T helper cells | 0.467 | ＜2.2E-16 |
| Regulatory T cells | 0.844 | ＜2.2E-16 |
| Activated B cells | 0.650 | ＜2.2E-16 |
| Immature B cells | 0.737 | ＜2.2E-16 |
| Memory B cells | 0.146 | 0.00467 |
| Natural killer cells | 0.497 | ＜2.2E-16 |
| CD56bright natural killer cells | 0.346 | 8.29E-12 |
| CD56dim natural killer cells | 0.253 | 8.13E-07 |
| Myeloid derived suppressor cells | 0.807 | ＜2.2E-16 |
| Natural killer T cells | 0.665 | ＜2.2E-16 |
| Activated dendritic cells | 0.698 | ＜2.2E-16 |
| Plasmacytoid dendritic cells | 0.420 | ＜2.2E-16 |
| Immature dendritic cells | 0.445 | ＜2.2E-16 |
| Macrophage | 0.755 | ＜2.2E-16 |
| Eosinophil | 0.384 | 1.59E-14 |
| Mast cells | 0.718 | ＜2.2E-16 |
| Monocyte | 0.342 | 1.55E-11 |
| Neutrophil | 0.421 | ＜2.2E-16 |

**Supplementary Table.6 Spearman correlation analysis between expression of LILRB5 and TILs in liver cancer (TISIDB)**

| Tumor-infiltrating lymphocytes | R value | P value |
| --- | --- | --- |
| Activated CD8+ T cells | 0.402 | ＜2.2E-16 |
| Central memory CD8+ T cells | 0.358 | 1.4E-12 |
| Effector memory CD8+ T cells | 0.590 | ＜2.2E-16 |
| Activated CD4+ T cells | 0.041 | 0.434 |
| Central memory CD4+ T cells | 0.121 | 0.0197 |
| Effector memory CD4+ T cells | 0.328 | 1.12E-10 |
| T follicular helper cells | 0.455 | ＜2.2E-16 |
| γδT cells | 0.425 | ＜2.2E-16 |
| Type 1 T helper cells | 0.503 | ＜2.2E-16 |
| Type 17 T helper cells | 0.161 | 0.00183 |
| Type 2 T helper cells | 0.181 | 0.00046 |
| Regulatory T cells | 0.557 | ＜2.2E-16 |
| Activated B cells | 0.417 | ＜2.2E-16 |
| Immature B cells | 0.472 | ＜2.2E-16 |
| Memory B cells | 0.236 | 4.48E-06 |
| Natural killer cells | 0.372 | 1.57E-13 |
| CD56bright natural killer cells | 0.226 | 1.08E-05 |
| CD56dim natural killer cells | 0.085 | 0.102 |
| Myeloid derived suppressor cells | 0.461 | ＜2.2E-16 |
| Natural killer T cells | 0.368 | 2.98E-13 |
| Activated dendritic cells | 0.324 | 1.77E-10 |
| Plasmacytoid dendritic cells | 0.313 | 7.76E-10 |
| Immature dendritic cells | 0.372 | 1.38E-13 |
| Macrophage | 0.522 | ＜2.2E-16 |
| Eosinophil | 0.477 | ＜2.2E-16 |
| Mast cells | 0.491 | ＜2.2E-16 |
| Monocyte | 0.294 | 8.55E-09 |
| Neutrophil | 0.291 | 1.21E-08 |
